# GlycoTraitR: an R package for characterizing structural heterogeneity in N-linked glycoproteomics data

**DOI:** 10.1093/bioadv/vbag244

**Published:** 2026-08-24

**Authors:** Bingyuan Zhang, Koichi Himori, Yusuke Matsui

**Affiliations:** Institute for Glyco-core Research (iGCORE), Nagoya University, Furo-cho, Chikusa-ku, Nagoya, Aichi 464-8601, Japan; Institute for Glyco-core Research (iGCORE), Nagoya University, Furo-cho, Chikusa-ku, Nagoya, Aichi 464-8601, Japan; Institute for Glyco-core Research (iGCORE), Nagoya University, Furo-cho, Chikusa-ku, Nagoya, Aichi 464-8601, Japan; Biomedical and Health Informatics Unit, Department of Integrated Health Science, Nagoya University Graduate School of Medicine, Nagoya, Aichi 461-8671, Japan

## Abstract

**Motivation:**

Glycoproteomics data are rapidly accumulating owing to advances in mass spectrometry and specialized search engines that identify N-linked glycopeptide spectrum matches together with glycan structures. However, the structural heterogeneity of glycosylation remains difficult to interpret, and no unified trait-based framework analyzes these data at both glycosylation-site and protein levels.

**Results:**

We developed GlycoTraitR, an R package for trait-based analysis of structural heterogeneity in N-linked glycoproteomics data. It imports search-engine outputs, extracts biologically interpretable glycan structural traits, quantifies microheterogeneity and macroheterogeneity, and compares heterogeneity across experimental conditions.

**Availability and implementation:**

The package and source code are freely available at https://github.com/matsui-lab/glycoTraitR. Documentation and a quick-start guide are available at https://matsui-lab.github.io/glycoTraitR/.

## 1 Introduction

Recent advances in both mass-spectrometry instrumentation and computational methods have substantially expanded the depth and reliability of glycoproteomics (Khoo 2021).

Modern search engines such as pGlyco3 and Glyco-Decipher further increase confident N-linked glycopeptide spectrum matches (GPSM) through “glycan-first” strategies with rigorous false-discovery-rate (FDR) control. However, N-linked GPSM data presents several statistical challenges in the downstream analysis. First, the combination of N-glycans and peptides leads to a large feature space when analyzed at the glycoform level. Second, GPSM is usually sparse, as many glycopeptides are unobserved or missing due to the stochastic nature of the data dependent acquisition (DDA). Third, structurally related glycans carried by the same peptide are not independent observations but share structural similarity and compositional features. There is a clear need for tools that fully leverage the structural information encoded in the attached glycans and peptides. As a result, the field lacks a reproducible, systematic framework for analyzing N-linked glycoproteomics data.

We focus on a fundamental yet often under-analyzed feature of glycoproteins: their intrinsic heterogeneity. Glycoprotein heterogeneity occurs at two levels (Potel *et al*. 2025). Macroheterogeneity refers to the diversity of glycan structures attached across the glycosylation sites of a protein, whereas microheterogeneity captures structural variation among glycoforms at a single site. These heterogeneous glycoforms influence protein folding, stability, receptor binding, and downstream signaling. Large-scale glycoproteomics studies have demonstrated that such site-specific heterogeneity is extensive and reproducible, and that glycan composition patterns and site-specific glycan ratio changes can sensitively capture biologically relevant variation (Potel *et al*. 2025, Wu *et al*. 2026, Zhu *et al*. 2025).

However, there is no analytical framework for quantifying and testing heterogeneity change in N-linked glycoproteomics data. Recent trait-based approaches developed in the glycomics field—such as glycowork (Thomès *et al.* 2021), glycompare (Bao *et al.* 2021), and glytrait (Fu *et al*. 2025)—demonstrate that analyzing glycan substructures can reveal meaningful biological patterns. However, most existing methods focus primarily on glycan composition or glycan-derived traits and do not explicitly account for glycoform heterogeneity. Although glycowork includes a generalized linear model framework to identify site-specific changes in glycan composition between experimental groups, it does not model changes in glycoform heterogeneity. Consequently, there is currently no unified trait-based framework that integrates both glycan structural traits and the peptide backbone to systematically investigate glycoform heterogeneity changes in glycoproteomics datasets. To address this gap, we developed a new analytical framework for quantifying heterogeneity directly from N-linked GPSMs, enabling interpretable trait-level comparisons at both the site and protein levels. We implemented this framework in the R package GlycoTraitR, which integrates with pGlyco3 and Glyco-Decipher outputs and supports trait extraction, differential analysis, visualization, and user-defined motifs for flexible, biologically driven analyses.

## 2 GlycoTraitR overview

GlycoTraitR implements a workflow that transforms N-linked glycopeptide spectrum matches (GPSMs) into quantitative measures of glycan structural heterogeneity at both the glycosylation-site and protein levels. As summarized in Fig. 1, the pipeline takes GPSM files from glycoproteomics search engines, performs abundance normalization, extracts structural traits for each N-glycan, computes microheterogeneity and macroheterogeneity scores, and applies a unified permutation-based statistical test to compare heterogeneity across experimental conditions. Site-level (micro) and protein-level (macro) heterogeneity analyses are computed in parallel by default, so that both levels of structural heterogeneity are reported for every trait without requiring the user to specify a level.

**Figure 1:**
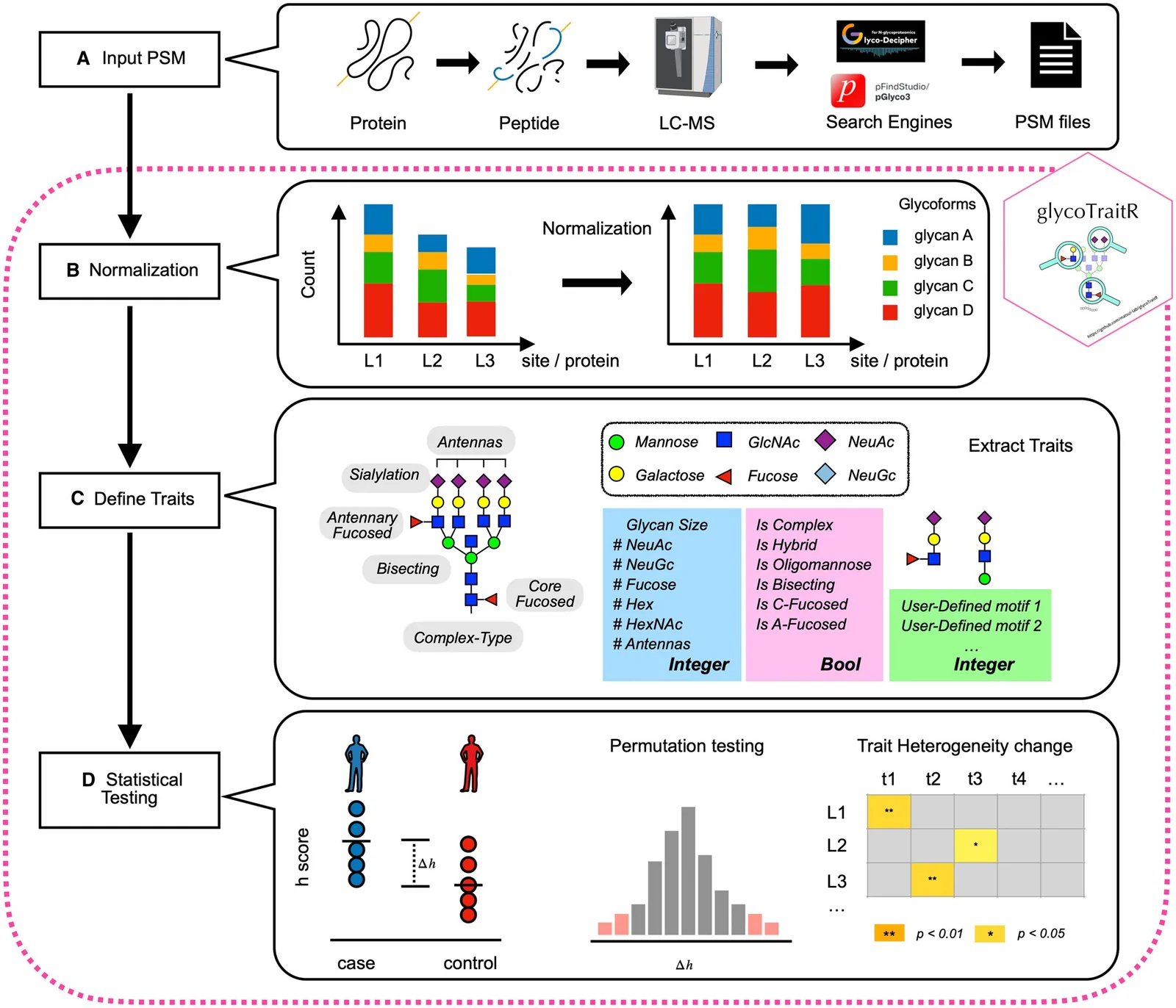
Overview of the GlycoTraitR workflow. (A) LC—MS/MS glycoproteomics experiments generate peptide spectra that are searched with glycoproteomics engines such as pGlyco3 and Glyco-Decipher, yielding N-linked glycopeptide spectrum matches (GPSM) with assigned glycan structures. (B) Within each glycosylation site or protein, the raw GPSM counts of individual glycoforms are converted to relative abundances summing to one, removing sample-to-sample variation in library size and making glycoform compositions directly comparable across samples. (C) For each distinct N-glycan, GlycoTraitR parses the structure and decomposes it into a panel of compositional and structural traits. Moreover, user-defined glycan motifs (UD-motif) can be specified as additional traits. (D) For every (trait, feature) pair, sample-level heterogeneity scores are compared between two experimental groups using the group-mean difference as the test statistic (left). A two-sided permutation p-value is obtained by random shuffling of the group labels, taking both tails of the null distribution (middle; shaded bars). Significant changes are summarized in an output table. The procedure is applied in parallel to site-level (micro) and protein-level (macro) heterogeneity.

### 2.1 Input

GlycoTraitR accepts a table of N-linked GPSMs generated from LC–MS/MS data and processed by glycoproteomics search engines that report glycan structures, such as pGlyco3 or Glyco-Decipher. The table contains a unique GPSM identifier, sample label, protein accession, glycosylation-site, glycan-structure string, and a numeric spectral-count column used to quantify each GPSM (Fig. 1A). In addition, metadata linking sample labels to group labels is required.

### 2.2 Sample-level normalization

GlycoTraitR normalizes the spectral count of each glycoform into a relative abundance within the corresponding site or protein (Fig. 1B). In particular, the site-level relative abundance is defined as the proportion of a glycoform detected at the same site, and the protein relative abundance is defined as the proportion of a glycoform among all GPSMs of the same protein. Both quantities sum to one, and are invariant to overall library size, making the downstream trait comparison independent of the sample difference. See supplementary for full definitions.

### 2.3 Trait definition

For each assigned N-glycan, GlycoTraitR parses the reported glycan string and decomposes it into a predefined set of structural traits that capture biologically interpretable aspects of glycan composition and architecture. The parser directly reuses glycan annotations produced by the search engines, enabling consistent trait extraction without redefining glycan structures (Fig. 1C). Both composition-based and structure-based traits are supported, allowing glycan heterogeneity to be represented at the trait level rather than as individual glycoforms. In addition to the built-in trait set, GlycoTraitR allows users to define custom glycan motifs as user-defined traits. These motifs are evaluated for each GPSM and incorporated into subsequent analyses, enabling flexible, hypothesis-driven interrogation of specific glycan substructures. See an example of the extracted traits of an N-glycan in Supplementary Information (Fig. S1, available at supplementary material  *Bioinformatics Advances* online).

### 2.4 Heterogeneity scoring and statistical testing

Using the normalized abundance and extracted traits, we compute heterogeneity scores per sample for every trait, quantifying how variable a trait is across the glycoforms of a site or protein (Fig. 1D). Both heterogeneity scores are defined in a unified mathematical form as abundance-weighted variances, and this definition also applies uniformly to integer and Boolean traits. By default, we apply a permutation-based two-sample test by randomly shuffling group labels 1000 times and recalculate the trait heterogeneity scores to generate the null distribution. The permutation procedure is distribution-free and applies to both integer and Boolean traits. We have retained the two-sample *t*-test as an optional method in the package. In situations where the assumptions of the *t*-test are approximately satisfied, the *t*-test is computationally faster and may be more statistically robust and efficient. Results are summarized as a table of proteins and sites whose heterogeneity changes across groups. See the definitions of the heterogeneity scores, permutation procedure and an application case on real data in Supplementary Information, available at supplementary material  *Bioinformatics Advances* online.

## 3 Discussion

In this work, we present a trait-based framework for quantifying glycan structural heterogeneity in N-linked glycoproteomics data. By decomposing assigned N-glycans into biologically interpretable structural traits and aggregating these traits at the glycosylation-site and protein levels, GlycoTraitR enables systematic evaluation of heterogeneity across experimental conditions. This representation shifts the analytical focus from individual glycopeptide-level comparisons to trait-level patterns, facilitating scalable and interpretable analyses of complex glycoproteomics datasets. As a demonstration, a real-data case study on Alzheimer’s disease brain glycoproteomics—processed with both pGlyco3 and Glyco-Decipher—is provided in the Supplementary Information (Figs S2 and S3, available at supplementary material  *Bioinformatics Advances* online).

The current implementation relies on discrete N-glycan structures reported by glycoproteomics search engines such as pGlyco3 and Glyco-Decipher and uses GPSM counts as quantitative measures. Consequently, the framework inherits the inherent trade-offs of tandem mass spectrometry–based structure assignment, where identification depth, structural resolution, and confidence must be balanced. As additional search engines begin to report explicit and standardized glycan structures, trait-based frameworks such as GlycoTraitR will support more systematic cross-study comparisons and integrative analyses. As future work, it can be extended to take intensity-based quantification output as the input once the quantification output with glycan structures becomes available for N-glycoproteomics search engines.

The predefined trait library in GlycoTraitR focuses on monosaccharide composition and canonical architectural features, while support for user-defined motifs allows flexible extension toward study-specific or functionally motivated substructures. As functional annotations and biosynthetic knowledge of N-glycans continue to expand, the trait space can be further enriched with topology-aware or data-driven features. In this context, GlycoTraitR provides a practical and extensible foundation for trait-centered interrogation of N-glycosylation heterogeneity.

## Supplementary Material

vbag244_Supplementary_Data

## Data Availability

The processed data underlying this article are available in Zenodo at https://zenodo.org/records/21386154. The GlycoTraitR source code is available at https://github.com/matsui-lab/glycoTraitR.
