# Supplementary material for: GlycoTraitR: an R package for characterizing structural heterogeneity in N-linked glycoproteomics data
Source: Bioinform Adv. 2026 Aug 24;6(1):vbag244. doi: 10.1093/bioadv/vbag244 (PMC13624273; doi:10.1093/bioadv/vbag244)
Supplement: Supplementary file 1 [file vbag244_supplementary_data.zip › GlycoTraitR_Supplementary_final.docx]

**Supplementary material of**

**GlycoTraitR: an R package for characterizing structural heterogeneity in N-linked glycoproteomics data**

Bingyuan Zhang^1^, Koichi Himori^1^, Yusuke Matsui^1,2*^

1. Institute for Glyco-core Research (iGCORE), Nagoya University, Nagoya, Aichi, Japan.
2. Biomedical and Health Informatics Unit, Department of Integrated Health Science,

Nagoya University Graduate School of Medicine, Nagoya, Japan

**Abstract**
In this supplementary, we introduce the definition of default traits, the definition of heterogeneity, the permutation-based statistical testing, and a case study applying the glycoTraitR package to Alzheimer’s disease brain glycoproteomics data. These results demonstrate that trait-based glycan representation enables robust and biologically meaningful comparison of N-glycosylation patterns.

**Default Trait Definition**

GlycoTraitR includes a set of predefined traits that capture a glycan's compositional and structural features. These traits are derived by parsing the monosaccharide composition and structure of the glycan string from either WURCS 2.0 or pGlyco3 format. The default trait library is designed to convert different glycoforms into common biologically interpretable descriptors, enabling quantification of N-glycan heterogeneity.

The default traits fall into two categories: (i) integer traits and (ii) Boolean traits. This representation follows established and known characterizations of N-glycans, including glycan class (oligomannose, hybrid, complex), branching degree, core fucosylation, antenna fucosylation, and bisecting N-acetylglucosamine. Together, these integer and Boolean traits provide a compact yet expressive representation of each N-glycan for downstream quantitative analyses. The default traits are defined as follows:

*Integer traits*

- Glycan size: Total number of monosaccharide residues.
- HexNAc: Number of N-acetylhexosamine residues.
- Hex: Number of hexose residues (mannose or galactose).
- Fucose: Number of fucose residues.
- NeuAc: Number of N-acetylneuraminic acid residues.
- NeuGc: Number of N-glycolylneuraminic acid residues.
- Antennas: Represents the number of terminal branches extending from the Man3GlcNAc2 core. We define an antenna as a branch that originates from one of the mannose residues extending outward from the core and is initiated by an N-acetylglucosamine (GlcNAc) residue.

*Boolean traits*

- IsComplex: A glycan is defined as a complex-type N-glycan when it has two or more branches extending from the central mannose residues Man1GlcNAc2, and these branches are elaborated with N-acetylglucosamine units rather than mannose alone.
- IsHybrid: A glycan is defined as a hybrid N-glycan when containing features of both oligomannose and complex-type structures: at least one branch retains a mannose-only extension, while another branch is extended with N-acetylglucosamine or other complex-type residues.
- IsOligomannose: A glycan is considered oligomannose when it contains multiple mannose residues extending beyond the conserved Man3GlcNAc2 core, and all outward branches arising from the central mannose unit consist exclusively of mannose.
- IsBisecting: A glycan with a bisecting N-acetylglucosamine (GlcNAc) residue attached to the central mannose (Man1GlcNAc2).
- IsC-Fucosed: A glycan with a core fucosylation.
- IsA-Fucosed: A glycan with at least an antenna fucosylation.

These traits provide a compact yet expressive representation of an N-glycan that is suitable for quantitative comparisons. User-defined motifs are treated as integer-valued traits, and their values correspond to the number of times each motif occurs within the glycan structure.

*Example:*

We provide an example of how glycoTraitR parses an N-glycan into traits. The glycan (G42295MA) from glycosmos (Yamada et al., 2020) is shown in Figure S 1.

Figure S 1. The example N-glycan (G42295MA)

The following is the wurcs 2.0 format of the glycan, and we demonstrate how to extract its structural traits using glycoTraitR functions.

```
# WURCS representation of the glycan
wurcs <- "WURCS=2.0/7,12,11/
 [a2122h-1x_1-5_2*NCC/3=O][a2122h-1b_1-5_2*NCC/3=O]
 [a1122h-1b_1-5][a1122h-1a_1-5][a2112h-1b_1-5]
 [Aad21122h-2a_2-6_5*NCC/3=O][a1221m-1a_1-5]
 /1-2-3-4-2-5-6-2-4-2-5-7
 /a4-b1_a6-l1_b4-c1_c3-d1_c4-h1_c6-i1_d2-e1_e4-f1_f6-g2_i2-j1_j4-k1"

glycan_tree <- glycoTraitR:::wurcs_to_tree(wurcs)
glycoTraitR::compute_glycan_traits(glycan_tree, motifs = NULL)

GlycanSize    Hexose    HexNAc    Neu5Ac    Neu5Gc   Fucose
    12           5         5        1         0         1
Antennas   IsBisecting    IsComplex    IsOligomannose    IsHybrid
     2           1            1               0              0
IsC-Fucosed   IsA-Fucosed
     1             0
```

**Input GPSM data and Normalization**

Let sample be indexed by $i=1, \ldots, n,$ protein by $j=1,\ldots, p,$ site by $k=1, \ldots, s,$ and glycan by $l=1,\ldots,g$. Let $c_{i,j,k,l}$ denote the GPSM count of glycoform $l$ at site $k$ of protein $j$ in sample $i$.

The relative glycoform abundance at site level is:

$$a_{i,j,k,l}:=\frac{c_{i,j,k,l}}{\sum_{l} c_{i,j,k,l}}$$

i.e. the proportion of glycoform $l$ among all glycoforms detected at site $\left( j,k \right)$. Therefore, $\sum_{l} a_{i,j,k,l}=1$, and the relative abundance becomes independent of sample library size and become comparable across samples.

Similarly, the relative glycoform abundance at protein $j$ is:

$$A_{i,j,l}:=\frac{\sum_{k} c_{i,j,k,l}}{\sum_{k,l} c_{i,j,k,l}}$$

i.e. the proportion of glycoform $l$ at protein $j$. Note that $\sum_{l} A_{i,j,l}=1$.

**Definition of heterogeneity**

Following the above definition of relative abundance, we clarify the explicit definition of micro- and macro-heterogeneity at each site or protein level. We use $t_{l}^{m}$ to denote the integer and Boolean traits $m=1, \ldots, t$ can be extracted from a glycan $l$.

The micro-heterogeneity score of trait $m$ for a glycosylated site (protein $j$, site $k$) in sample $i$ is defined as follows:

$$h_{i,j,k}^{m}:= \sum_{l} a_{i,j,k,l}\left( t_{l}^{m}-\mu_{i,j,k}^{m} \right)^{2}, \mu_{i,j,k}^{m}:=\sum_{l} a_{i,j,k,l}*t_{l}^{m},$$

where $\mu_{i,j,k}^{m}$ is the mean trait abundance across the glycoforms detected at site $k$.

The macro-heterogeneity score of trait $m$ at glycoprotein $j$ in sample $i$ is defined as follows:

$$H_{i,j}^{m}:= \sum_{l} A_{i,j,l}\left( t_{l}^{m}-\mu_{i,j}^{m} \right)^{2}, \mu_{i,j}^{m}:=\sum_{l} A_{i,j,l}*t_{l}^{m},$$

where $\mu_{i,j}^{m}$ is the mean trait abundance across the glycoforms detected at protein $j$.

For a given protein, the number of contributing glycosylation sites is the same across the compared groups, so differences in site number do not confound the resulting p-value. Because the number and structural context of glycosylation sites vary across proteins, macro-heterogeneity scores are not directly comparable between different proteins. Caution is also warranted when the set of detected sites differs between groups for the same protein (e.g., due to missing data), as this can affect the site-weighted mixture underlying the macro-heterogeneity score.

We note that both micro- and macro heterogeneity are defined as weighted variances and therefore take positive values. The same definition applies uniformly to different types of integer-valued and Boolean traits. For Boolean traits, the formulas can be further simplified:

$$h_{i,j,k}^{m}=\mu_{i,j,k}^{m}\left( 1-\mu_{i,j,k}^{m} \right), H_{i,j}^{m}=\mu_{i,j}^{m}\left( 1-\mu_{i,j}^{m} \right),$$

as the weighted variance collapse to the Bernoulli variance. The Bernoulli form also makes explicit that, for a Boolean trait, trait abundance and trait heterogeneity are functionally coupled: knowing $\mu$ fully determines heterogeneity.

**Connection between micro/macro-heterogeneity**

To build an intuitive understanding of the connection between micro- and macro- heterogeneity, we decompose the protein-level score $H_{i,j}^{m}$ into its within-site and between-site components. We start by noting that the protein-level glycoform abundance $A_{i,j,l}$ can be expressed as a site-weighted mixture of the corresponding site-level abundances:

$$A_{i,j,l}= \sum_{k} w_{i,j,k}a_{i,j,k,l}$$

where

$$w_{i,j,k}:=\frac{\sum_{l} c_{i,j,k,l}}{\sum_{k,l} c_{i,j,k,l}} , \sum_{k} w_{i,j,k}=1,$$

is the relative GPSM contribution of site $k$ to the total of protein $j$. Substituting this identity into the definition of $H_{i,j}^{m}$ and expanding the squared deviation, the macroheterogeneity score has the closed-form decomposition:

$$H_{i,j}^{m}= \sum_{k} w_{i,j,k}h_{i,j,k}^{m}+ \sum_{k} w_{i,j,k}\left( \mu_{i,j,k}^{m}- \mu_{i,j}^{m} \right)^{2}$$

which is a simple insight by decomposing the total variance under our weighting scheme. However, the decomposed two terms have direct biological interpretations:

1. The first term is the site-weighted average of microheterogeneity score over the glycosylation sites of the protein.
2. The second term is the weighted variance of site-level mean trait values across the glycosylation sites of the protein. It captures how trait profiles differ between sites — the classical "macro" notion in glycobiology.

Therefore, we find the definition straight forward.

**Permutation-based two sample testing**

To test whether trait-wise micro- or macro-heterogeneity differs between two experimental groups (e.g., case vs. control), we adopt a permutation-based two-sample procedure. The test is distribution-free and applies identically to Boolean and integer traits, providing a unified statistical framework for the heterogeneity scores. Let $G_{\text{case}}$ and $G_{\text{ctrl}}$ denote the sets of samples in the two groups, with sizes $n_{\text{case}}$and $n_{\text{ctrl}}$, and let $n=n_{\text{case}}+n_{\text{ctrl}}$. For trait $m$ and feature (site $\left( j,k \right)$or protein $j$), the group-mean heterogeneity scores are

$$\bar{h}_{G,j,k}^{m}:=\frac{1}{\mid G\mid}\sum_{i\in G} h_{i,j,k}^{m}, \bar{H}_{G,j}^{m}:=\frac{1}{\mid G\mid}\sum_{i\in G} H_{i,j}^{m}.$$

where $G\in\{G_{case}, G_{ctrl}\}$. The test statistic $T$ is defined as the difference $\Delta h_{j,k}^{m}$ or $\Delta H_{j}^{m}:$

$$\Delta h_{j,k}^{m}:= \bar{h}_{G_{case}, j,k}^{m}- \bar{h}_{G_{ctrl}, j,k}^{m}, \Delta H_{j}^{m}:= \bar{H}_{G_{case}, j}^{m}- \bar{H}_{G_{ctrl}, j}^{m}$$

We need to generate the null distribution, which is approximated by label permutation. We randomly permute group labels $B$ times and recompute the test statistic $T^{b}$ under the permutated labels, which yields the empirical null distribution $\{T{\}}_{b=1}^{B}$. The p-value is given by

$$p=\frac{1+\sum_{b=1}^{B} \boldsymbol{1}\left( \left| T^{b} \right|\geq\left| T \right| \right)}{1+B}$$

Where $\boldsymbol{1}\left( \boldsymbol{\cdot} \right)$ is an indicator function. This permutation-based testing is distribution-free, meaning that no distributional assumptions are required for $h_{i,j,k}^{m}$ or $H_{i,j}^{m}$. Moreover, it can be applied to both Boolean and integer traits. Unless otherwise specified, we used B = 1,000 label permutations.

**Case Study**

Next, we showcase and apply glycoTraitR to a publicly available dataset. The case study follows the complete workflow in glycoTraitR, including data import, trait derivation, trait–GPSM matrix construction, statistical testing, and visualization.

**Data**

The case study is based on the intact N-glycoproteomics dataset (Suttapitugsakul et al., 2022). The full dataset is publicly available under the ProteomeXchange identifier *PXD032219*. It consists of LC-MS/MS measurements of enriched N-glycoproteins from postmortem human frontal cortex tissues representing three groups:

i. individuals with no cognitive impairment and no pathological AD,
ii. asymptomatic AD subjects (pathology present without cognitive decline), and
iii. symptomatic AD subjects.

The HILIC-enriched glycopeptide runs from *PXD032219* was used. The raw LC-MS/MS files were re-analyzed using both pGlyco3 and Glyco-Decipher with the following setting to obtain glycopeptide spectral matches (GPSMs). Descriptive statistics of the study cohort and glycoproteomics dataset are summarized in Supplementary Table S1. All processed output GPSM files and Table S1 are archived in Zenodo [https://zenodo.org/records/21386154](https://zenodo.org/records/17759790) to ensure the reproducibility.

**Search engine settings**

The raw files were searched using both Glyco-Decipher (version 1.0.5) and pGlyco3 (version 3.1). All searches were performed using the default parameters recommended by each search engine for intact N-glycopeptide identification. Glyco-Decipher searches employed the built-in N-glycan database containing 10,936 glycan entries, while pGlyco3 searches used the *pGlyco3-N-Human* glycan library (2,922 human N-glycans). The human proteome database *UP000005640* downloaded from UniProt on March 26, 2025. Both search engines used the same UniProtKB human reference proteome as the protein sequence database. The resulting glycopeptide spectral matches (GPSMs) from both engines were processed for subsequent analysis using glycoTraitR <https://github.com/matsui-lab/glycoTraitR>.

**GlycoTraitR analysis**

The analysis of the example above consisted of four steps:

1. importing the glycopeptide spectral matches (GPSMs);
2. preprocessing the GPSMs by collapsing technical replicate runs into a single sample-level dataset through merging identical GPSMs and summing their spectral counts.
3. using the wrapper function to compute per-sample micro- and macro-heterogeneity scores at the site and protein levels in parallel and to perform two-sample tests evaluating heterogeneity differences between groups;
4. visualizing the trait change in two ways (an heatmap and a volcano plot).

Below we provide representative R code that illustrates the core steps used in this case study. In addition, we compared the traits that exhibited significant changes in both the pGlyco3 and Glyco-Decipher GPSM datasets and highlight the commonly altered traits together with a brief discussion of their potential biological implications.

*Import GPSM file(s)*

```
library(glycoTraitR)

# Preprocess the pGlyco3 GPSM matrix
gpsm_dir <- "/path_to_your_file/pGlycoDB-GP-FDR-Pro-Quant-Site.txt"
pGlyco3_gpsm <- read_pGlyco3_gpsm(gpsm_dir)

# Preprocess the GlycoDecipher GPSM folder
gpsm_folder_dir <- "/path_to_your_folder/"
decipher_gpsm <- read_decipher_gpsm(gpsm_folder_dir)

# Load sample metadata (a data frame)
meta <- readRDS("/path_to_your_file/meta.rda")
head(meta)

# A tibble: 6 × 22
  Diagnosis     `Sample number` `Tissue weight (mg)` `Age at baseline`
  <fct>                    <dbl>                <dbl>             <dbl>
1 Asymptomatic                1                    62              77.1
2 Normal                    110                    72              70.8
3 Normal                    118                    74              75.7
4 Normal                     12                    57              80.8
5 Normal                    125                    52              89.2
6 Normal                    127                    24              85.2
# 18 more variables: `Age at death` <dbl>, `APOE gene` <chr>,
#   Braak <dbl>, CERAD <dbl>, `Global Cog Func` <chr>,
#   Reagan <dbl>, arteriol_scler <chr>, ci_num2_gct <dbl>,
#   ci_num2_mct <dbl>, cvda_4gp2 <chr>, dlbdx <dbl>, Sex <chr>,
#   hspath_any <dbl>, Race <chr>, tdp_cs_6reg <chr>,
#   tdp_stage4 <chr>, PMI <dbl>, file <chr>
```

Import GPSM data and sample meta. The package supports GPSM tables generated by either pGlyco3 or GlycoDecipher. First, read the GPSM results and prepare a sample metadata table containing sample identifiers and experimental group information. The metadata table must include a `File` column matching the sample names in the GPSM table.

*Collapsing technical replicate runs*

```
library(dplyr)

pGlyco3_gpsm$File <- pGlyco3_gpsm$File |>
  sub("^KS_AD170(?:I|II|III)_HILIC-elu_[0-9]+_[0-9]+$",
      "KS_AD170_HILIC-elu", x = _) |>
  sub("^KS_AD363(?:I|II|III)_HILIC-elu_[0-9]+_[0-9]+$",
      "KS_AD363_HILIC-elu", x = _)

pGlyco3_gpsm <- pGlyco3_gpsm |>
  group_by(across(-Count)) |>
  summarise(Count = sum(Count), .groups = "drop")

decipher_gpsm$File <- decipher_gpsm$File |>
  sub("^KS_AD170(?:I|II|III)_HILIC-elu_[0-9]+_[0-9]+$",
      "KS_AD170_HILIC-elu", x = _) |>
  sub("^KS_AD363(?:I|II|III)_HILIC-elu_[0-9]+_[0-9]+$",
      "KS_AD363_HILIC-elu", x = _)

decipher_gpsm <- decipher_gpsm |>
  group_by(across(-Count)) |>
  summarise(Count = sum(Count), .groups = "drop")
```

The example dataset contains technical replicate runs for two biological samples (KS_AD170 and KS_AD363). Before downstream analysis, these replicate runs were collapsed into a single sample by merging identical GPSMs while summing their spectral counts. This preprocessing step ensures that each biological sample is represented only once in the subsequent glycosylation trait analysis.

*Evaluate heterogeneity differences between groups*

```
set.seed(2026)
res_pGlyco3 <- analyze_hscore_changes(
  gpsm = pGlyco3_gpsm,
  from = "pGlyco3",
  motifs = NULL,
  meta = meta,
  group_col = "Diagnosis",
  group_levels = c("Normal", "Symptomatic"),
  B = 1000,
  min_samples = 3
)

res_decipher <- analyze_hscore_changes(
  gpsm = decipher_gpsm,
  from = "decipher",
  motifs = NULL,
  meta = meta,
  group_col = "Diagnosis",
  group_levels = c("Normal", "Symptomatic"),
  B = 1000,
  min_samples = 3
)
```

Here, analyze_hscore_changes() performs differential analysis of glycan trait abundance (mean) and heterogeneity score between two sample groups. The function computes glycan traits from a GPSM table, summarizes them at both site and protein levels, and evaluates group differences using permutation-based testing. Group label is defined by group_col and group_levels, while B controls the number of permutations used to estimate p-values. Features with insufficient observations in either group are excluded according to min_samples.

*Visualization*

plot_hscore_heatmap(res_decipher)

The heatmap summarizes significant changes in glycosylation trait abundance (μ) and heterogeneity scores (h) between groups at both the protein and glycopeptide levels. Color intensity reflects the direction and magnitude of changes, while asterisks indicate statistical significance. Only the significant changes between groups are shown here.

plot_hscore_volcano(res_decipher)

We also provide another volcano plot to highlight glycosylation trait changes between groups. Each point represents a protein- or site-level trait, with effect size on the x-axis and statistical significance on the y-axis, enabling identification of the most altered features.

*Result*

Several protein-specific glycosylation alterations were observed in symptomatic subjects. NCAM2 exhibited a decrease in Oligomannose glycans accompanied by an increase in complex-type glycans, suggesting enhanced glycan maturation on this synaptic adhesion molecule, which has been implicated in neuronal connectivity and Alzheimer's disease (Haukedal & Freude, 2020; Zhang et al., 2024). PTGDS, another protein extensively studied in the context of AD, showed a reduction in glycan size heterogeneity, indicating a shift toward a more homogeneous glycosylation state that would not be captured by conventional mean-abundance analyses alone. At the site-specific level, NPTN displayed reduced bisecting glycan structures, whereas NFASC exhibited both decreased antennary abundance and increased antennary heterogeneity. Collectively, these results demonstrate that GlycoTraitR provides complementary insights from a glycan-trait perspective. GlycoTraitR identified coordinated remodeling of glycan maturation, branching, and structural diversity at protein and site level.

**Reference**

Haukedal, H., & Freude, K. K. (2020). Implications of glycosylation in Alzheimer’s disease. *Frontiers in Neuroscience*, *14*, 625348.

Suttapitugsakul, S., Stavenhagen, K., Donskaya, S., Bennett, D. A., Mealer, R. G., Seyfried, N. T., & Cummings, R. D. (2022). Glycoproteomics landscape of asymptomatic and symptomatic human Alzheimer’s disease brain. *Molecular & Cellular Proteomics: MCP*, *21*(12), 100433.

Yamada, I., Shiota, M., Shinmachi, D., Ono, T., Tsuchiya, S., Hosoda, M., Fujita, A., Aoki, N. P., Watanabe, Y., Fujita, N., Angata, K., Kaji, H., Narimatsu, H., Okuda, S., & Aoki-Kinoshita, K. F. (2020). The GlyCosmos Portal: a unified and comprehensive web resource for the glycosciences. *Nature Methods*, *17*(7), 649–650.

Zhang, Q., Ma, C., Chin, L.-S., Pan, S., & Li, L. (2024). Human brain glycoform coregulation network and glycan modification alterations in Alzheimer’s disease. *Science Advances*, *10*(14), eadk6911.
